# Graphitic Carbon Nitride Nanoheterostructures as Novel Platforms for the Electrochemical Sensing of the Chemotherapeutic and Immunomodulator Agent MTX

**DOI:** 10.3390/bios13010051

**Published:** 2022-12-29

**Authors:** Reham G. Elfarargy, Mahmoud A. Saleh, Mohamed Mahrous Abodouh, Mahmoud A. Hamza, Nageh K. Allam

**Affiliations:** Energy Materials Laboratory, School of Sciences and Engineering, The American University in Cairo, New Cairo 11835, Egypt

**Keywords:** graphitic carbon nitride, electrochemical sensors, methotrexate, LOD, DFT

## Abstract

We report on the electrochemical determination of one the most effective and widely used chemotherapeutic, anti-inflammatory, and immunomodulator agents, methotrexate (MTX), using low-cost, green, and facile one-pot prepared graphitic carbon nitride (g-CN ) nanosheets. The g-CN nanosheets have been characterized utilizing Fourier transform infrared spectroscopy, X-ray diffraction(XRD), scanning electron microscopy(SEM), and density functional theory (DFT). In comparison to the bare carbon paste electrode (CPE), the g-CN -modified electrode showed a spectacular enhancement in the electrochemical oxidation and detection abilities of MTX. The proposed material exhibits very low limits of detection (12.45 nM) and quantification (41.5 nM), while possessing a wide linear range of 0.22–1.11 μM and 1.11–27.03 μM under optimized conditions at pH 7.0. Due to the ease of preparation of g-CN, it can be adopted for the cost-effective detection of MTX in industrial and clinical analyses.

## 1. Introduction

Methotrexate (MTX) (*N*-(4-{[(2,4-diaminopteridin-6-yl) methyl] (methyl)amino}benzoyl)-L-glutamic acid) is an antineoplastic [1,2] and immunomodulator [1,3,4] therapeutic agent. MTX has a plethora of uses, such as treating a variety of cancers and inflammatory reactions including breast cancer [5,6,7], lung cancer [8,9,10], leukemia [8,11,12], lymphoma [13,14,15], and osteosarcoma [16]. In addition, it possesses high efficacy against some autoimmune disorders, including rheumatoid arthritis [17,18], psoriasis [19], Crohn’s disease [20], and sarcoidosis [21]. MTX possesses two main actions that depend on different mechanisms. The anticancer, immunomodulatory, and anti-inflammatory reaction of MTX includes many sub-mechanisms, such as the inhibition of dihydrofolate reductase which antagonizes folic acid production in tumor cells and rapidly dividing lymphocytes, the inhibition of adenosine intracellular catabolism (which is considered a potent anti-inflammatory mediator), the inhibition of polyamine synthesis by inhibiting tetrahydrofolate and methylhydrofolate (which are mediators for lymphotoxins in rheumatoid arthritis), and the synthesis of reactive oxygen species that induce cell death in T-cells and suppress cytokine production [22].

The need to monitor MTX emerges from its narrow therapeutic index [23] and fatal adverse effects that contradict its exceptional history of treatment and range of doses. Its adverse effects include kidney toxicity [24,25], hepatotoxicity [26,27], bone marrow suppression [28,29], tumor lysis syndrome [30], and increased risk of infection due to suppression of immunity [31], which necessitates the therapeutic drug monitoring of MTX. Until recently, techniques such as high-performance liquid chromatography, turbulent flow liquid chromatography, and ultraviolet-visible absorption spectrometry have been utilized to determine the concentration of MTX [32,33,34,35]. These approaches have limited practical platform use due to their difficult processes, high costs, and time-consuming nature. In general, the electrochemical approach demonstrated outstanding real-time measurement, cost-effectiveness, ease of use, and sensitivity in the detection of clinical samples, which improved the practicality of the detection of MTX [36]. The electrochemical determination of MTX has been established in several studies. The materials explored include metal oxides, carbon nanodots, carbon nanotubes, and different composites. However, most of the reported platforms are either based on expensive materials or require very tedious processing.

Herein, we demonstrate the effective use of graphitic carbon nitride (g-CN) as an electrode modifier. In addition to being a low-cost material, g-CN can be synthesized via environmentally friendly routes at large scales [37,38]. Moreover, g-CN has attracted extensive attention for being the most stable carbon nitride allotrope [39]. Additionally, it is thermally and chemically stable due to its high condensing temperature with high electrocatalytic activity [39]. Hence, it is implemented in the fabrication of sensitive electrochemical sensors [40]. Motivated by those characteristics, we report on the use of g-CN to analyze MTX electrochemically with a very low limit of detection (LOD) and very wide range of linearity. Density functional theory (DFT) calculations were used to elucidate the involved mechanism.

## 2. Experimental

### 2.1. Materials

Urea was purchased from Alfa Aesar (Ward Hill, MA, USA) and was purified extra through recrystallization in absolute ethyl alcohol. Ethylenediaminetetraacetic acid disodium and polyvinylidene difluoride powder (PVDF) were also obtained from Alfa Aesar. Sulfuric acid (H_2_SO_4_) and dimethylformamide (DMF) were purchased from Sigma-Aldrich (Darmstadt, Germany). Hydrochloric acid (HCl) was purchased from Honeywell (Morristown, NJ, USA). Carbon black and graphite sheets with 0.3 mm thickness were obtained from Xinruida, (Shanghai, China). All the materials used were reagents of analytical grade without any further purification. Deionized water (DI) was prepared by an ultrapure water system using Millipore Direct-Q3 with UV from Merck (Boston, MA, USA).

### 2.2. Synthesis of Graphitic Carbon Nitride

According to the procedure described in our prior publication [41], g-C_3_N_4_ nanosheets were created using a simple one-pot method based on the thermal polymerization of urea. Briefly, the extra-purified urea was poured into a porcelain crucible that had been lined with commercial aluminum foil and then into the entire reaction pot. This system underwent a 3-h, 550 °C air annealing process in a muffle furnace at a 10 °C/min heating rate. To remove any excess ammonia, the obtained yellowish powder was ultrasonically dispersed for 1 h in 0.1 M HNO_3_ at room temperature. The powders were then filtered, thoroughly cleaned with distilled water, and dried at 80 °C.

### 2.3. Sensor Fabrication

**Scheme 1:**
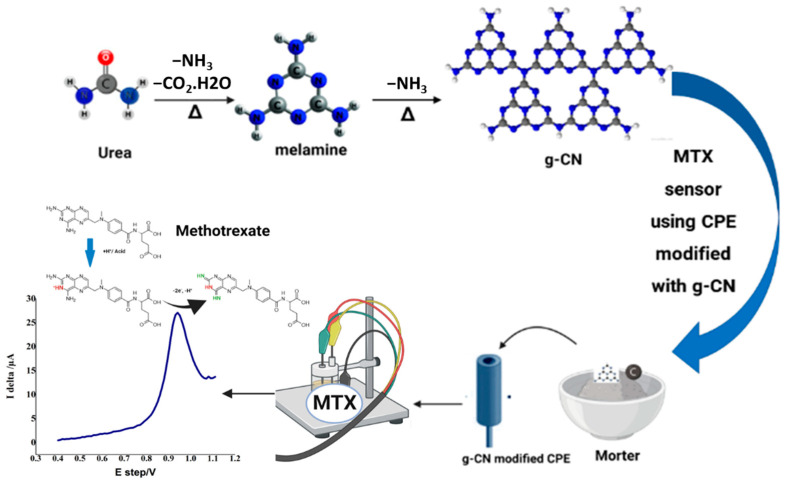
Stepwise synthesis and testing of the sensor as well as the proposed mechanism of electrochemical oxidation of Methotrexate (MTX).

By uniformly combining 1.0 g of graphite powder and 0.27 mL of paraffin oil in a small mortar, the bare carbon paste electrode (CPE)was created. The paste was then carefully packed into the electrode’s cavity. Before use, the CPE’s surface was polished on a fresh piece of paper. Prior to scanning, the CPE was submerged in the supporting electrolyte. The paste was emptied, renewed, and polished following each scan. By combining 1.0 g of graphite with 15 mg of g-CN and homogenising the mixture for two minutes with a spatula, the modified electrode was created. To create the paste, 0.27 mL of paraffin was also added and mixed for 45 min. The paste was packed and regenerated as previously mentioned. The characterization and computational procedures are included in the Supplementary File. The procedure is summarized in Scheme 1.

### 2.4. Analysis of Real Samples

Plasma. Fresh plasma samples were provided for the tests by a healthy person. By adding 10 mL of the supernatant to 9 mL of pH 7 Britton–Robinson buffer (BRB), MTX stock solution concentration was obtained. The solution was then, without further pretreatment, transferred to an electrochemical cell for analysis.

Methotrexate^®^. Five Methotrexate^®^ 2.5 mg tablets were weighed and ground to prepare a stock solution containing 1 mM of MTX. To 25.0 mL of ethanol, the calculated quantity of the powder containing a specified amount of drug is added. Hence, 25.0 mL of ethanol was added to the flask and mixed with a vortex mortar to dissolve the added amount. After filtering the solution through 0.45-m filter paper, the 1 mM of MTX stock solution was prepared for further analysis.

Urine. Fresh urine samples were taken from a healthy person prior to the experiments. The desired concentration was achieved by adding various concentrations of MTX stock solution to 10 L of supernatant that had been spiked to 9 mL of pH 7 BRB. Without applying any additional pretreatment, the solution was transferred to an electrochemical cell for analysis.

## 3. Results and Discussion

### 3.1. Morphological, Structural, Compositional, and Surface Characteristics of g-CN

**Figure 1:**
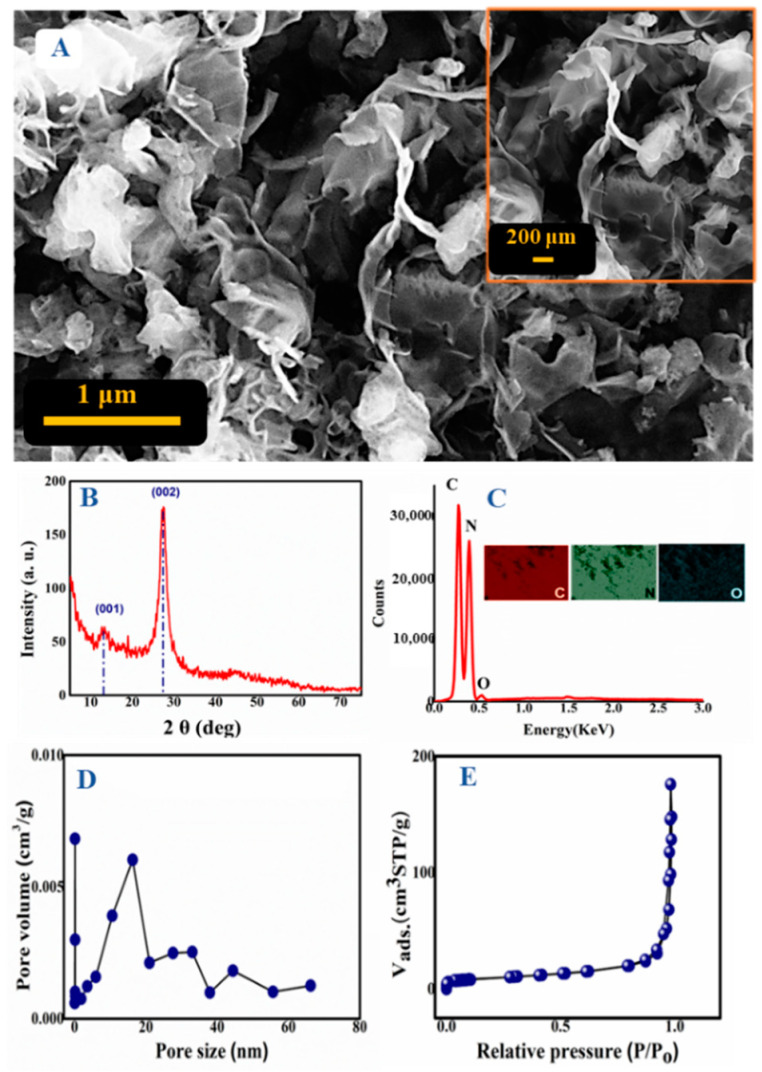
(**A**) Field emission scanning electron microscopy (FESEM) images with high and low magnification, (**B**) Energy Dispersive X-Ray (EDX) spectra and elemental mapping, (**C**) X-ray diffraction (XRD) spectra, (**D**) Brunauer-Emmett-Teller (BET) adsorption-desorption isotherm, and (**E**) pore size distribution of the prepared graphitic carbon nitride (g-CN).

The morphology of the prepared g-CN nanosheets was investigated using Field emission scanning electron microscopy (FESEM) as shown in Figure 1A, revealing the formation of layered nano-sheets. Additionally, the elemental composition of the nanosheets was investigated using Energy Dispersive X-Ray Analysis (EDX) analysis as shown in Figure 1B, demonstrating the coexistence of C, N, and O. The EDX mapping reveals the homogeneous distribution of the elements with no foreign elements detected, indicating the high purity of the fabricated 2D g-CN nanosheets. The crystal structure of the fabricated g-CN nanosheets was elucidated via X-ray diffraction (XRD) analysis. The XRD spectra showed two diffraction peaks (Figure 1C). The peak at 13.3° can be indexed to the (100) plan, which is a characteristic of the interlayer stacking of conjugated aromatic systems [42,43] as indicated by the interplanar distance (d-spacing) of the main peak of 3.25 Å. The second peak at 27.4° can be ascribed to the repeated in-plane heptazine units. In addition, High-Resolution X-ray photoelectron spectroscopy (HR-XPS) analysis was used to determine the chemical composition of the g-CN nanosheets. In consonance with the obtained EDX data, the HR-XPS survey spectrum (Figure S1a) confirms the presence of C, O, and N only, without any impurities. The HR-XPS C 1s spectrum in Figure S1b exhibits two prominent peaks, at 284.6 and 288.0 eV, that are attributed to C-N=C and C-single bond C, respectively. In addition, Figure S1c depicts the HR-XPS N 1s spectrum, which can be decomposed into four primary peaks. Pyridinic nitrogen (at 398 eV), which is connected to an aromatic ring by a sp^2^-bond, replaced one carbon atom in the aromatic ring. As a result of this alteration, the electronic characteristics of the graphite layer are improved, and the electrochemical oxidation of the carbon surface is prevented. While the other, less distinct peaks at 399.1, 400.9, and 404.1 eV, respectively, are for tertiary nitrogen (N single bond C), amino nitrogen (N single bond H), and nitrogen-oxide groups. It is noteworthy that the estimated nitrogen content of the g-CN powder surpasses 12%. The HR-XPS O 1s spectrum in Figure S1d can be deconvoluted into two sub-peaks at 532.6 and 531.81 eV, which are attributed to the oxygen-carbon bond and adsorbed water molecules, with a ratio of 26.7:73.3, respectively.

The nitrogen adsorption/desorption isotherm of the synthesized g-CN nanosheets is shown in Figure 1D, demonstrating a typical IV type isotherm (mesoporous material) with an H3 hysteresis loop (slit-like pores). The Brunauer-Emmett-Teller (BET) specific surface area of the g-CN is 32.51 m^2^/g. In addition, the Barrett-Joyner-Halenda (BJH) model was used to determine the total pore volume and average pore diameter, as shown in Figure 1E. The calculated total pore volume and average pore diameter are 0.30 cm^3^/g and 1.868, respectively. 

### 3.2. DFT Characterization of the g-CN

**Figure 2:**
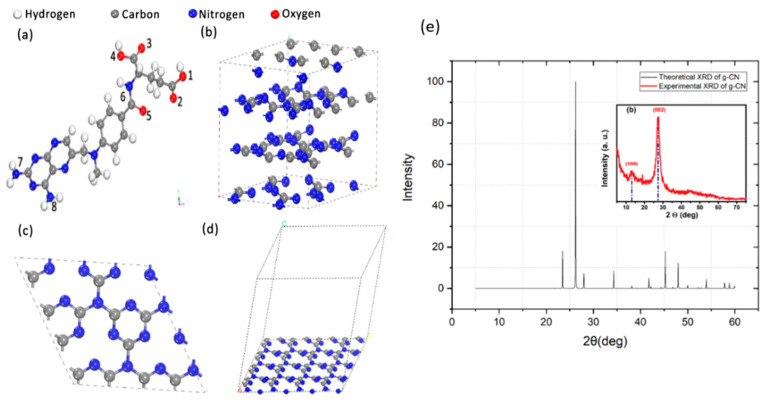
Structure of (**a**) MTX with identical positions of (1, 4), (2, 3), and (7, 8), (**b**) g-CN (Bulk), (**c**) Facet (002) cleaved from the bulk g-CN, (**d**) surface (002) with vacuum slap, and (**e**) theoretical versus experimental XRD spectra of g-CN.

**Figure 3:**
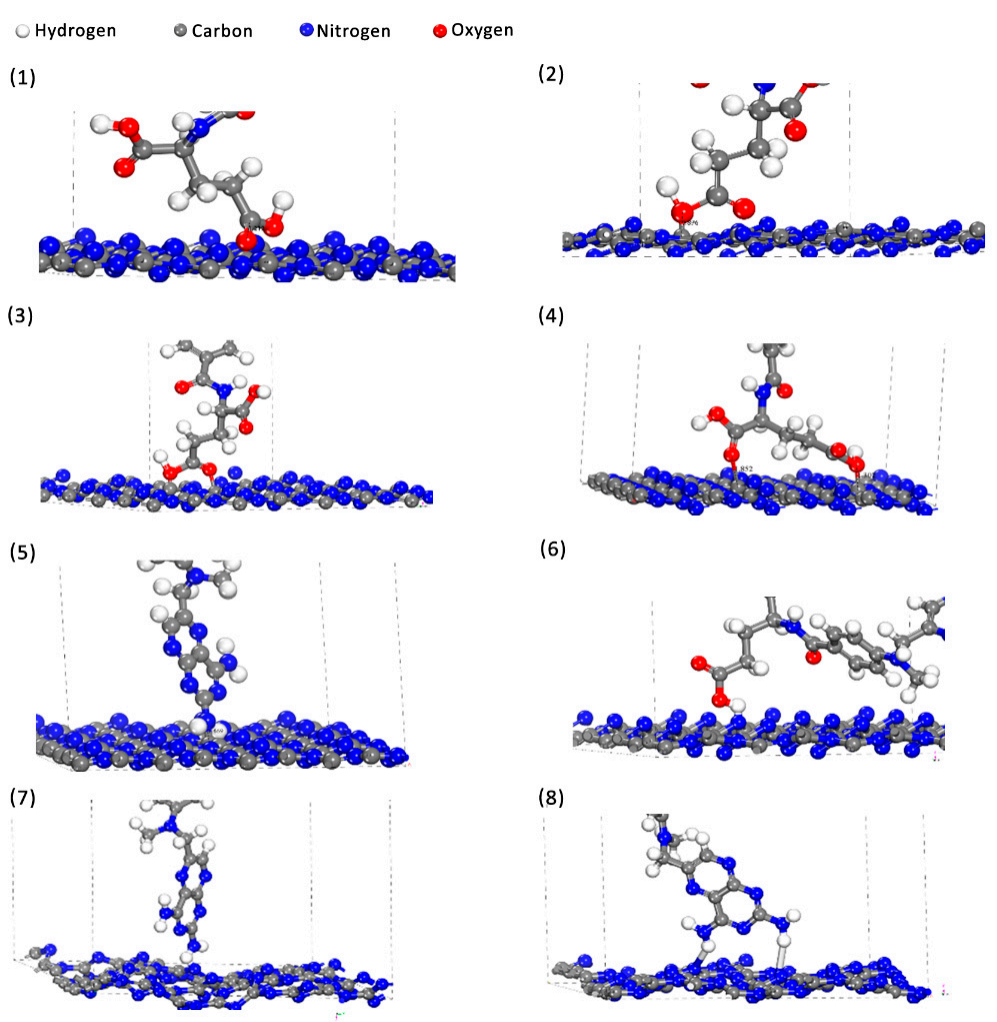
Adsorption positions of the (**1**) Carbonyl group on the carbon atom, (**2**) Hydroxyl group on the carbon atom, (**3**) Carboxylic group on the carbon atom, (**4**) Carbonyl group and hydroxyl group on the carbon atom, (**5**) Amino group on the carbon atom, (**6**) Hydrogen atom of the hydroxyl group on the Nitrogen atom, (**7**) Hydrogen atom of the Amino group on the Nitrogen atom, and (**8**) two Hydrogen atoms of the Amino group on the carbon atom.

The structures of the MTX and g-CN bulk (Figure 2) were left to relax for geometry optimization until reaching their lowest formation energy. To validate the structure of g-CN, a theoretical XRD profile has been generated using a REFLEX module implemented in Materials Studio software and compared to its experimental profile [44] (Figure 2e). Both spectra showed the same peak positions with a dominant (002) plan. In addition, the calculated (2.3 eV) and experimentally reported electronic band gaps (2.7 eV) [45] are very similar. To build the most dominant surface, which is the (002) facet, a 2 × 2 × 2 supercell of g-CN formed the 2D plane (Figure 2) after cleaving the bulk structure, then a vacuum slap of 25 Å was formed to avoid the interactions between the atoms of the cleaved surface and its periodic planes. For all calculations, DMOL3 module was used, and the generalized gradient approximation (GGA) was applied with the Perdew−Burke−Ernzerhof (PBE) exchange-correlation functional in the convergence criteria of 1.0 × 10^−5^ Ha, 0.002 Ha/Å and 0.005 Å for energy, force, and displacement tolerance, respectively, through a Monkhorst−Pack k-point sampling of (2 × 2 × 2) [46]. As MTX has various functional groups and all these groups could be available for adsorption on the g-CN surface, the charge on each atom was calculated for both absorbent and adsorbate, as shown in Table 1. As expected, it has been found that all functional groups (Oxygen and Nitrogen atoms) of the MTX molecule have negative charges. However, the distribution of charge values over the g-CN surface was negative for nitrogen atoms and positive for carbon atoms. The difference in charges means that MTX cannot be adsorbed via its functional groups with negative charges on the Nitrogen atoms, due to the repulsion between the similar charges. As g-CN has the same type of carbon atoms in its structure, to determine which functional group would be adsorbed, the adsorption energy was calculated for each possible case via Equation (1) [46], as listed in Table 2 and depicted in Figure 3.

(1) $$E_{ads}=E_{Total}-E_{g-CN}-E_{MTX}$$

where *E_ads_*, *E_g-CN_*, *E_MTX_*, and *E_total_* are the energy of the adsorbed molecule, the total energy of the surface isolated, the total energy of the single adsorbate, and the total energy of the adsorbate on the adsorbent, respectively.

**Figure 4:**
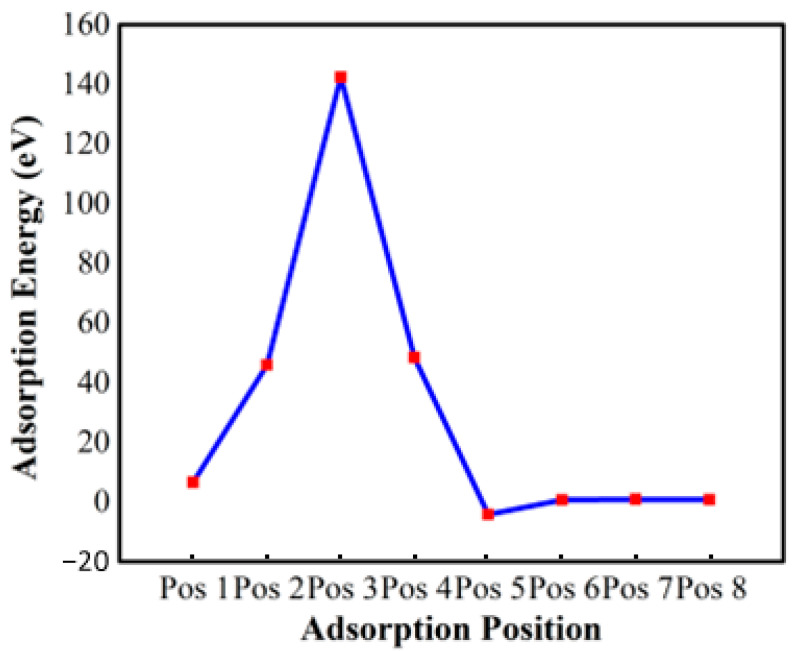
Adsorption energy as a function of the adsorption location.

Before setting the possible scenarios for the adsorption of MTX on g-CN, the possibility of adsorbing MTX via its central atoms was excluded due to the steric hindrance of its neighboring groups [47]. The total energy of formation for the MTX structure alone was found to be −43,221.72 eV, while for the (002) surface of g-CN, it was −145,010.37 eV. The 1–4 adsorption positions, where the oxygen atoms are attracted to the carbon atom of the g-CN surface, are not favorable due to their high adsorption energy (Table 1). The same is true for positions 6–8, where the hydrogen atoms of the hydroxyl group and amino groups of the di-amino pyridine ring are attracted to the nitrogen atoms on the surface, and their adsorption energy was found to be higher than position 5, where it shows the lowest adsorption energy (Figure 4).

The mechanism of adsorbing the amino group through its Nitrogen atom on the g-CN surface was due to the difference in the electronegativity between the two atoms, which caused the development of partial negative charges (∂−) on the Nitrogen of the amino group and partial positive charges (∂+) on the carbon atom of the g-CN, leading to the attraction between them. However, the electronegativity difference between Hydrogen and Nitrogen of the amino group was found to be 0.84 eV [48], which is higher than the electronegativity difference between the Hydrogen atom and the Carbon atom on the g-CN surface, which was 0.35 eV [48]. That difference enhanced the electrostatic force between Hydrogen and Nitrogen atoms on the surface, reducing the bond between the Hydrogen and the Nitrogen atoms in the Amino group. This bond breaking caused the release of an electron, which could be the reason behind the higher current, as will be discussed later.

### 3.3. Electrochemical Performance

**Figure 5:**
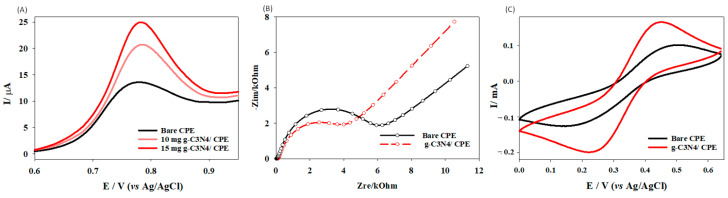
(**A**) Square-wave Voltammetry (SWV) of 1 × 10^−4^ M of MTX using bare carbon paste electrode (CPE), 10 mg and 15 mg of g-CN in pH 7.0 Britton–Robinson buffer (BRB), and (**B**,**C**) Electrochemical impedance spectroscopy (EIS) and Cyclic Voltammetry (CV) of bare CPE and g-CN in 5.0 × 10^−3^ M K_3_Fe(CN)_6_ in 0.1 M KCl, respectively, utilizing bare CPE and g-CN.

For the purpose of sensing MTX in Britton–Robinson (BR) buffer at 7.0 pH using 0.10 mM of MTX, square wave voltammetry (SWV) was used to examine the electrochemical behavior of bare against g-CN nanosheets-modified CPE platforms. In the absence of MTX, CPE there are no discernible anodic or cathodic peaks, indicating electrochemical inactivity of the sensor platform within the working-potential window. The anodic oxidation peak current of MTX on bare CPE was 13.61 μA at 0.78 V, which was dramatically increased upon the use of g-CN, reaching 24.96 μA at 0.78 V and demonstrating the simple oxidation of MTX. This means that the sensitive lower potential detection of MTX can be accomplished using g-CN /CPE. The highest electrochemical activity toward MTX, good conductivity, and a high rate of electron transfer have all been demonstrated for 15 mg of g-CN /CPE, as shown in Figure 5A. Upon the use of 5.0 × 10^−3^ M K_3_Fe(CN)_6_ in 0.10 M KCl and recording the current vs. voltage peak at different scan rates, the electroactive surface area of the prepared g-N/CPE sensor platform was calculated from the cyclic voltammograms using the Randles–Ševčík equation for a quasi-reversible reaction, Equation (2) [49]:

(2) $$I_{P}=2.65\times 10^{5}\;n^{\frac{3}{2}}\;AD^{\frac{1}{2}}\;Cv^{\frac{1}{2}}$$

where *A* is the electrode electrochemical surface area, *C* is the concentration of the redox probe, *D* is the diffusion coefficient, *I_p_* is the peak current, and *n* is the number of electrons involved in the electrochemical anodic oxidation. K_3_Fe(CN)_6_ has a *D* value of 7.6 × 10^−6^ cm^2^ s^−1^ [50]. The electrochemical surface area for bare CPE and g-CN /CPE was calculated to be 31.67 cm^2^ and 0.0506 cm^2^, respectively, using the slopes of the *I_p_* vs. v^1/2^ plot and Equation (2).

A 1:1 solution of 5.0 × 10^−3^ M K_3_Fe(CN)_6_ in 0.1 M KCl was utilized to examine the reaction kinetics, mass transport, and charge-transfer coefficient at the electrode surface using electrochemical impedance spectroscopy (EIS), as shown in Figure 5B. This can be accomplished via estimation of the charge-transfer resistance (R_CT_) at the surface of the electrode from the diameter of the quasi-circle in the high-frequency window. A typical Warburg-type equivalent circuit model can be obtained from the Nyquist plots. As a result, when using the proposed g-CN to modify the CPE, the charge transfer is superior using the bare CPE. When the bare CPE is modified with g-CN, the R_CT_ drops dramatically from 4400 to 2616.2 Ω, which can be attributed to the large surface area and interactive nature of g-CN that facilitates electron transfer. Additionally, Figure 5C shows a comparison between the electrochemical activity of the bare CPE and the g-CN/CPE electrode in a 1:1 solution of 5.0 × 10^−3^ M K_3_Fe(CN)_6_ in 0.1 M KCl. The g-CN/CPE electrode has an anodic peak current value that is nearly 1.5 times greater than that of bare CPE. Additionally, compared to the bare CPE, the use of the g-CN/CPE significantly reduced the peak separation (E_p_—E_p_) from 0.34 to 0.21 V, indicating enhanced electron transfer. Therefore, g-CN has good conductivity, a high rate of electron transfer, and good catalytic activity toward the electrochemical oxidation of MTX.

### 3.4. Effect of Scan Rate

**Figure 6:**
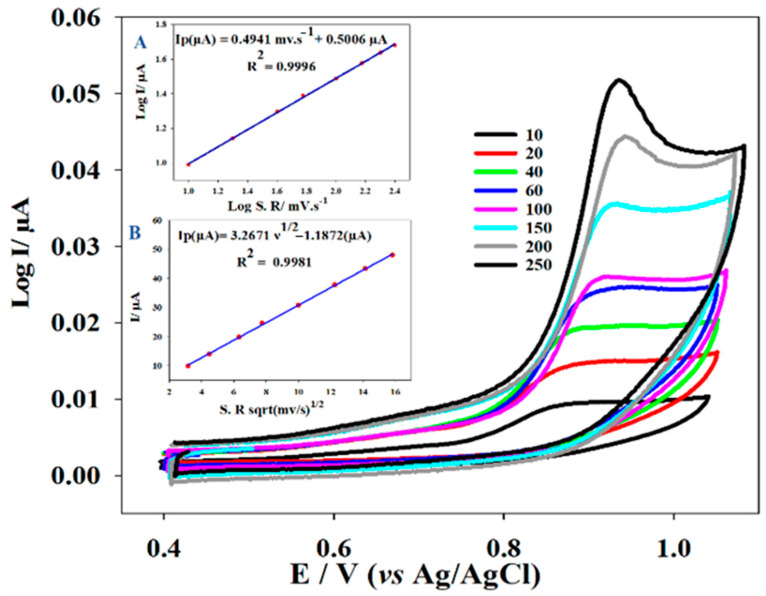
Cyclic voltammetry (CVs) of 0.10 mM MTX at pH 7.0 using the g-CN/CPE in the wide scan rate (0.010–0.250 V s^−1^) in pH 7.0 BRB. Insets: (**A**) plot of the log peak current vs log scan rate, and (**B**) plot of peak current vs the square root of the scan rate.

The effect of scan rate on the electrochemical anodic oxidation of MTX on g-CN /CPE is investigated using cyclic voltammetry, as shown in Figure 6. Upon increasing the scan rate, the peak potential (E_p_) is shifted into more positive values, indicating an irreversible electrochemical behavior of MTX. A linear relationship is observed, and the measured current is less than 0.5: *I_p_* (μA) = 0.4941 mVs^−1^ + 0.5006 µA, R^2^ = 0.9996, which indicates a diffusion-controlled process. Furthermore, the linear relationship between the peak current (*I_p_*) versus the square root of the scan rate (ν^1/2^) in the range of 0.010–0.250 V s^−1^. *I_p_* (μA) = 0.4941 mVs^−1^ + 0.5006 µA, R^2^ = 0.9996 also supports the idea that the anodic oxidation process is diffusion-controlled.

### 3.5. Analytical Performance Validation

**Figure 7:**
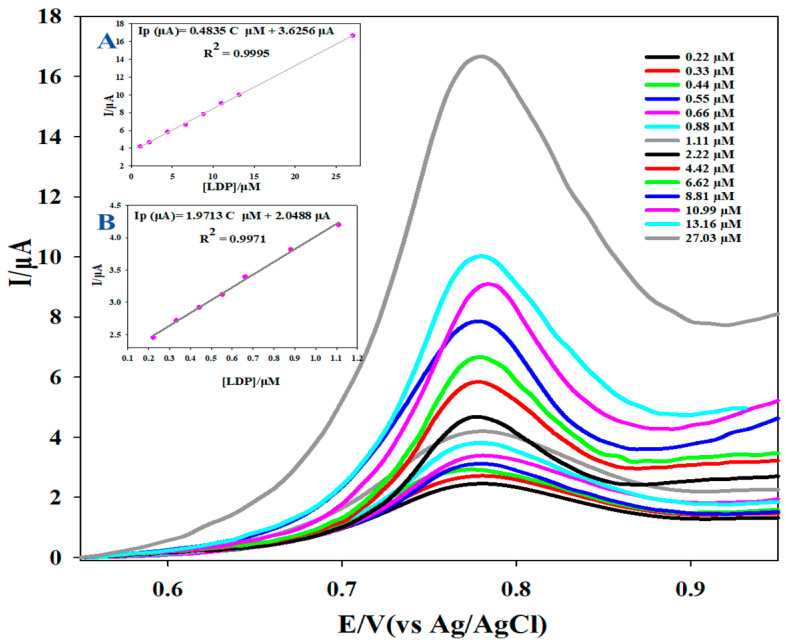
SWVs of several dilutions of MTX (27.03–0.22 μM) in pH 7.0 BRB using the g-CN/CPE sensor at a scan rate of 0.1 V s^−1^. The inset demonstrates the plot of the peak current as a function of concentrations in the range of (**A**) 0.22 × 10^−6^ to 1.109 × 10^−6^ and (**B**) 1.109 × 10–6 to 27.03 × 10^−6^ M.

For the validation of the proposed sensing protocol, the approved International Conference on Harmonization (ICH) guidelines for validating analytical methods were adopted in our study [51,52]. Square-wave Voltammetry (SWV) scans using the g-CN /CPE in BRB at pH 7.0 containing several dilutions of MTX were performed and analyzed. To achieve linearity, accuracy, and precision, the calibration curve is constructed to account for the practical range of MTX in the normal tablet concentration. The SWVs of these dilutions are shown in Figure 7. At a scan rate of 0.1 V s^−1^, MTX shows two linear behaviors based on its concentration in the BRB. In the range of 2.22 × 10^−7^ to 1.11 × 10^−6^ M, the regression equation is *I_p_* (μA) = 1.9713 C μM + 2.0488 μA, R^2^ = 0.9971, while in the range of 1.11 × 10^−6^ to 27.03 × 10^−6^ M, the regression equation is *I_p_* (μA) = 0.4872 C μM + 3.6038 μA, R^2^ = 0.9983. The main cause for the decrease in the slope of the second linear range at higher concentrations is the increase in the required energy for anodic stripping in addition to the Ohmic drop at such high levels of MTX. Additionally, this platform’s limit of detection (LOD) was calculated using the formula LOD = 3 SD/*x*, where SD is the standard deviation of the peak currents of MTX oxidation for *n* trials (*n* = 5) and *x* is the slope of the calibration curve. LOD was found to be 12.449 nM. Using the formula LOQ = 10 SD/*x* and the limit of quantification (LOQ) was determined to be 41.496 nM.

### 3.6. Analytical Application

#### 3.6.1. Analysis of Spiked Plasma Samples

**Figure 8:**
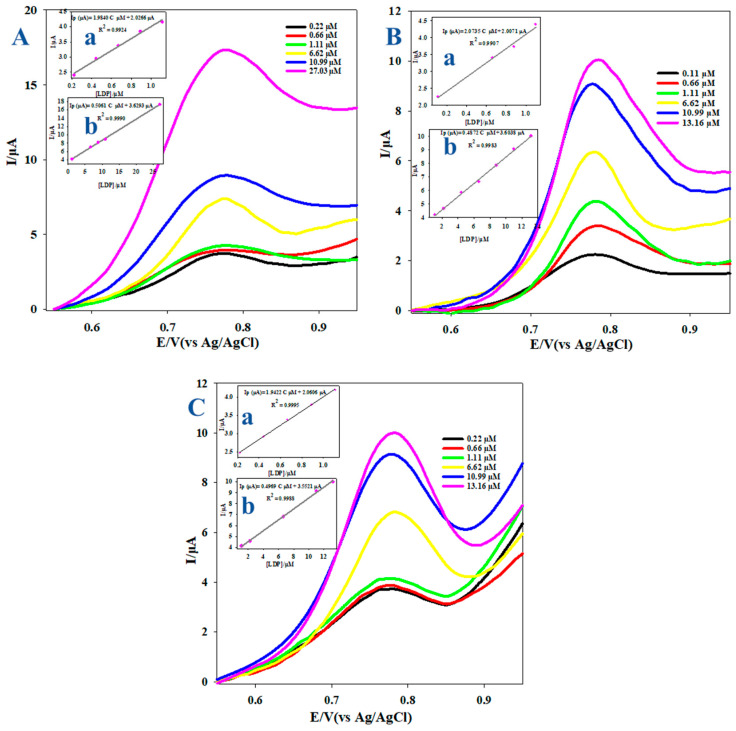
Square wave voltammogram for various concentrations of MTX in (**A**) spiked plasma (0.22–27.03 µM), (**B**) MTX Tablet (0.11–13.16 µM), and (**C**) spiked urine (0.22–13.16) samples in pH 7.0 of BR buffer at the scan rate of 100 mV s^−1^ using g-CN/CPE. Inset (**a**) shows the calibration curve of MTX with low concentrations of spiked plasma, tablets, and spiked urine. Inset (**b**) shows the calibration curve of MTX with high concentratios of spiked plasma, tablets, and spiked urine samples in pH 7 of BR buffer.

Utilizing SWV at a scan rate of 0.1 V s^−1^, MTX shows two linear behaviors based on its concentration in the BRB. In the range of 0.22 × 10^−6^ to 1.11 × 10^−6^ M, the regression equation is Ip (μA) = 1.9840 C μM + 2.0266 μA, R^2^ = 0.9924, while in the range of 1.11 × 10^−6^ to 27.03 × 10^−6^ M, the regression equation is *I_p_* (μA) = 0.5061 C μM + 3.6293 μA, R^2^ = 0.9990, as shown in Figure 8.

#### 3.6.2. Analysis of Methotrexate^®^ Tablet

Utilizing SWV at a scan rate of 0.1 V s^−1^, MTX shows two linear behaviors based on its concentration in the BRB. In the range of 0.11 × 10^−6^ to 1.11 × 10^−6^ M, the regression equation is Ip (μA) = 2.0735 C μM + 2.0071 μA, R^2^ = 0.9907, while in the range of 1.11 × 10^−6^ to 13.16 × 10^−6^ M, the regression equation is *I_p_* (μA) = 0.4872 C μM + 3.6038 μA, R^2^ = 0.9983, as shown in Figure 8.

#### 3.6.3. Analysis of Spiked Urine Samples

At a scan rate of 0.1 V s^−1^, MTX shows two linear behaviors based on its concentration in the BRB. In the range of 0.22 × 10^−6^ to 1.11 × 10^−6^ M, the regression equation is *I_p_* (μA) = 1.9422 C μM + 2.0606 μA, R^2^ = 0.9995, while in the range of 1.11 × 10−6 to 13.16 × 10^−6^ M, the regression equation is *I_p_* (μA) = 0.4969 C μM + 3.5521 μA, R^2^ = 0.9988.

Table 3 shows the electrochemical detection of various MTX concentrations using the conventional addition method. The recovery rates for MTX detection in dosage form and plasma samples show that the suggested platform has good precision, as could also be seen in Figure 8. The experimental results show that the sensor platform has excellent potential for the detection of MTX in both the dosage form and in urine or plasma samples.

### 3.7. Interference Studies

**Figure 9:**
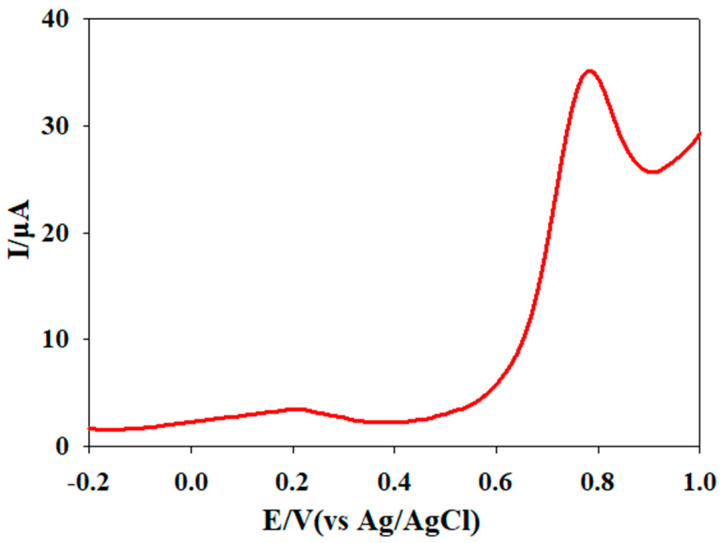
Square wave voltammogram of 0.1 mM MTX + 50 μM ascorbic acid in pH 7.0 BR buffer at a scan rate of 100 mV s^−1^ using g-CN /CPE.

The presence of ascorbic acid, which is regarded as an abundant molecule in the human body, was used to evaluate the selectivity of the suggested sensor platform to MTX. The use of the g-CN -modified electrode assisted in the peak differentiation of ascorbic acid peak. A square wave voltammogram of 50μM ascorbic acid (matching the highest level in plasma) [53] and 0.1 mM MTX showed distinct and spaced-out oxidation square wave voltammogram peaks at 0.2145 and 0.7805, respectively, at a scan rate of 100 mV s^−1^ (see Figure 9).

## 4. Comparison to Previous Studies

Due to the importance of monitoring MTX, it was extensively studied in the literature as shown in Table 4. In comparison to previous studies, our method showed outstanding linearity and LOD, while also being less expensive than other techniques.

## 5. Conclusions

In conclusion, this study demonstrates the preparation of g-CN for the detection and determination of MTX in buffer, spiked plasma, urine samples, and dosage form. The g-CN /CPE sensor platform showed the highest cost-efficiency among other published counterparts. In addition, DFT calculations helped to elucidate the oxidation mechanism. The proposed material showed limits of detection and quantification of 12.45 and 41.5 nM, respectively, while possessing a wide linear range of 0.22–1.11 μM and 1.11–27.03 μM under optimized conditions at pH 7.0, revealing the spectacular potential of the investigated sensor for use in a plethora of electrochemical applications.

## Figures and Tables

**Table 1:** The distribution of charges on each atom (position) in the MTX molecule.

Atom	Charge (eV)
O1	−0.401
O2	−0.383
O3	−0.391
O4	−0.424
O5	−0.480
N6	−0.391
N7	−0.358
N8	−0.368

**Table 2:** Formation and adsorption energy for each position via DMOL3 module.

Species	Formation Energy (eV)	Adsorption Energy (E_a_) (eV)
MTX (E_Adsorbate_)	−43,221.7222	−
g-CN (E_Adsorbent_)	−145,010.3784	−
Pos 1 (g-NC=O-MTX)	−188,225.5043	6.59633
Pos 2 (g-NC=OH-MTX)	−188,186.2121	45.8885
Pos 3 (g-NC=OH-MTX) & (g-NC=O-MTX) same group	−188,089.7114	142.3892
Pos 4 (g-NC=OH-MTX) & (g-NC=O-MTX) different group	−188,183.6613	48.4393
Pos 5 (g-NC=NH2-MTX)	−188,236.3192	−4.2185
Pos 6 (g-CN =HO-MTX)	−188,231.3822	0.7184
Pos 7 (g-CN =HNH-MTX)	−188,231.1617	0.9390
Pos 8 (g-CN =H2N-MTX)	−188,231.3344	0.7662

**Table 3:** Application of SWV for the determination of MTX in plasma, urine, and dosage form.

Pharmaceutical Formulations	Amount Added (µM)	Amount Found (µM)	Recovery%
**Methotrexate® (2.5 MG/TAB)**	0.11	0.12	104.99
	0.66	0.67	100.61
	0.88	0.83	93.84
	1.11	1.14	102.43
	4.42	4.42	99.80
	6.62	6.66	100.55
	10.99	10.76	97.94
	13.16	13.33	101.29
**Mean ± S.D.**	4.74 ± 0.05		
**Plasma**	0.22	0.23	101.34
	0.44	0.44	99.98
	0.67	0.66	99.10
	0.89	0.89	100.07
	1.11	1.11	100.25
	6.62	6.88	103.95
	8.81	9.05	102.75
	10.99	10.49	95.47
	27.03	27.09	100.24
**Mean ± S.D.**	6.32 ± 0.09		
**Urine**	0.22	0.22	100.26
	0.44	0.44	100.12
	0.67	0.68	101.46
	0.89	0.87	97.41
	1.11	1.12	101.12
	1.10	1.20	109.08
	2.22	2.09	94.23
	6.62	6.57	99.14
	10.99	11.25	102.38
	13.16	12.98	98.65
**Mean ± S.D.**	3.74 ± 0.05		

**Table 4:** Comparison between previous literature and our findings.

Method	Electrode	Modifier	pH	LWR	LOD	Application	Ref.
CV/EIS	GCE	Quaternary amine functionalized multi-wall carbon nanotubes (q-MWNTs)	5.0	0.022 to 44.01 μM	0.44 pM	Injection/tablet/urine/sample	[35]
CV/EIS	acetylene black paste electrode (ABPE)	acetylene black (AB) with stearyl trimethyl ammonium bromide (STAB)	6.0	0.005 μM to 7.0 μM	3.07 nM	Serum	[33]
CV/DPV	GCE	cerium-doped ZnO nanoflowers (Ce-ZnO/GCE)	7.0	0.01 to 500 μM	2.3 nM	Injection/spiked blood	[54]
CV/SWV	SPE	Au/MWNTs-ZnO/SPE	4.5	0.02 to 1.00 μM	10 nM	whole blood samples	[55]
CV/SWV	ITO (indium tin oxide)	biopolymers extracted from babassu mesocarp (BM)	3.5	0.5 to 5 0 μM	0.59 μM	-	[56]
CV/SWV/EIS	g-CN	CPE	7.0	0.22 × to 1.11 μM and 1.11 to 27.03	12.45 nM	Tablet/urine/sample	This study

## Data Availability

Data will be available upon request.

## Supplementary Materials

The following supporting information can be downloaded at: https://www.mdpi.com/article/10.3390/bios13010051/s1, Figure S1: HR-XPS spectra of the g-CN nanosheets: (a) survey, and the HR-XPS spectra of (b) C 1s, (c) N 1s, and (d) O 1s spectra; Physicochemical Characterization of g-CN; Computational details of density-functional theory (DFT); Sensor Fabrication.
