# Supplementary material for: Graphitic Carbon Nitride Nanoheterostructures as Novel Platforms for the Electrochemical Sensing of the Chemotherapeutic and Immunomodulator Agent MTX
Source: Biosensors (Basel). 2022 Dec 29;13(1):51. doi: 10.3390/bios13010051 (PMC9856071; doi:10.3390/bios13010051)
Supplement: Supplementary file 1 [file biosensors-13-00051-s001.zip › biosensors-2019703-supplementary.pdf]

1. Physicochemical Characterization of GCN

EDX measurements were done with an Oxford ISIS 310 spectroscopy. Panalytical X′pert PRO MPD X-ray diffractometer operating at 45 kV and 40 mA was used to record the XRD patterns with a Cu Kα radiation source (λ= 0.15418 nm, 2θ range = 5°–80°, scanning mode = continuous, step-size = 0.04°, and scan-step-time = 0.5 s). The elemental composition and surface oxidation state of the synthesized g-C_3N_4 powder were also determined using X-ray photoelectron spectroscopy (XPS). Using Quanta Chrome Nova 3200e, which has pore size and surface area analyzers, nitrogen adsorption and desorption isotherms were recorded at 77 K. After degassing all samples at 373 K overnight, all tests were performed as per the manufacturer's instructions. We calculated the specific surface area (m^2/g) of the investigated powders by Brunauer-Emmett-Teller (BET) analysis of the adsorption data. Additionally, the Barrett-Joyner-Halenda (BJH) model was used to estimate the pore size distribution of the samples. EDX spectroscopy (Oxford ISIS 310, England) was used to determine the composition of atoms within the matrix of the fabricated material and determine the distribution of atoms within it. a Panalytical X′pert PRO MPD X-ray diffractometer was used to determine the crystal structure through recording the XRD pattern and operating in a continuous mode at 45 kV and 40 mA, using an exposure source of Cu with a radiation source Kα (α = 0.154018 nm) within the 2θ range from 5° to 80°, with a scan step time of 0.5 s and a step size of 0.04°.

X-ray photoelectron spectroscopy was also used to determine the surface chemical composition of the generated active material (XPS using a Thermo-Scientific ESCALAB 250Xi spectrometer). In addition, we used Quanta Chrome Nova 3200e to determine nitrogen adsorption-desorption isotherms. Utilizing the Brunauer-Emmett-Teller (BET) relationship and Barrett-Joyner-Halenda (BJH) model to determine the surface area and pore size distribution of the fabricated material.

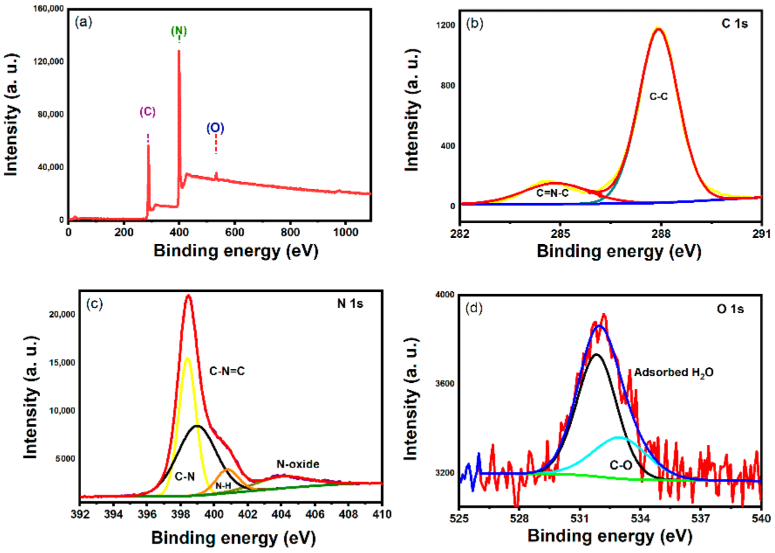

Figure S1. XPS spectra of the g-CN nanosheets: (**a**) survey, and the HR-XPS spectra of (**b**) C 1s, (**c**) N 1s, and (**d**) O 1s spectra.

2. Computational Details of Density-Functional Theory (DFT)

For a better understanding of the electrochemical measurements observed, Density Functional Theory (DFT) was used to simulate the adsorption process of MTX molecule on the surface of Graphitic carbon nitride G-CN to explain the reason behind the high peak of the Square Wave Voltammetry (SWV) appeared when MTX added on the surface

of Graphitic Carbon Nitride. Materials studio 7.0 software was used for building the structure of G-CN and MTX, and to calculate the energy of formation via the DMOL3 module, which is an effective and accurate tool for explaining the adsorption process for surfaces.

3. Sensor Fabrication

The CPE was prepared by mixing 1.0 g of graphite powder and 0.27 mL of paraffin oil uniformly by grinding in a small mortar. Then, a small amount of the paste is packed into the cavity of the electrode. The surface of the CPE is smoothed by polishing on a clean paper before its use. The CPE was immersed in the supporting electrolyte prior to scanning. After each scan, the paste is emptied, regenerated, and polished. The modified electrode was prepared by mixing 1.0 g of graphite with 15 mg of g-CN, and the mixture is homogenized by mixing with a spatula 2 min. Furthermore, 0.27 mL of paraffin was added and mixed for 45 min to obtain the paste. The paste is packed and regenerated as aforementioned.
